# Snoring-related polygenic risk and its relationship with lifestyle factors in a Korean population: KoGES study

**DOI:** 10.1038/s41598-023-41369-x

**Published:** 2023-08-30

**Authors:** Borim Ryu, Sejoon Lee, Eunjeong Heo, Sooyoung Yoo, Jeong-Whun Kim

**Affiliations:** 1 Center for Data Science, Biomedical Research Institute, Seoul Metropolitan Government-Seoul National University Boramae Medical Center, Seoul, Republic of Korea; 2 Precision Medicine Center, Seoul National University Bundang Hospital, Seongnam, Republic of Korea; 3 Healthcare ICT Research Center, Office of eHealth Research and Businesses, Seoul National University Bundang Hospital, 172, Dolma-ro, Bundang-gu, Seongnam-si, Gyeonggi-do 13605 Republic of Korea; 4 Department of Otorhinolaryngology, Seoul National University Bundang Hospital, 172, Dolma-ro, Bundang-gu, Seongnam-si, Gyeonggi-do 13605 Republic of Korea; 5 Department of Otorhinolaryngology, Seoul National University College of Medicine, Seoul, Republic of Korea

**Keywords:** Medical genomics, Biomarkers, Genetics research

## Abstract

Whereas lifestyle-related factors are recognized as snoring risk factors, the role of genetics in snoring remains uncertain. One way to measure the impact of genetic risk is through the use of a polygenic risk score (PRS). In this study, we aimed to investigate whether genetics plays a role in snoring after adjusting for lifestyle factors. Since the effect of polygenic risks may differ across ethnic groups, we calculated the PRS for snoring from the UK Biobank and applied it to a Korean cohort. We sought to evaluate the reproducibility of the UK Biobank PRS for snoring in the Korean cohort and to investigate the interaction of lifestyle factors and genetic risk on snoring in the Korean population. In this study, we utilized a Korean cohort obtained from the Korean Genome Epidemiology Study (KoGES). We computed the snoring PRS for the Korean cohort based on the UK Biobank PRS. We investigated the relationship between polygenic risks and snoring while controlling for lifestyle factors, including sex, age, body mass index (BMI), alcohol consumption, smoking, physical activity, and sleep time. Additionally, we analyzed the interaction of each lifestyle factor and the genetic odds of snoring. We included 3526 snorers and 1939 nonsnorers from the KoGES cohort and found that the PRS, a polygenic risk factor, was an independent factor for snoring after adjusting for lifestyle factors. In addition, among lifestyle factors, higher BMI, male sex, and older age were the strongest lifestyle factors for snoring. In addition, the highest adjusted odds ratio for snoring was higher BMI (OR 1.98, 95% CI 1.76–2.23), followed by male sex (OR 1.54, 95% CI 1.28–1.86), older age (OR 1.23, 95% CI 1.03–1.35), polygenic risks such as higher PRS (OR 1.18, 95% CI 1.08–1.29), drinking behavior (OR 1.18, 95% CI 1.03–1.35), late sleep mid-time (OR 1.17, 95% CI 1.02–1.33), smoking behavior (OR 0.99, 95% CI 0.82–1.19), and lower physical activity (OR 0.92, 95% CI 0.85–1.00). Our study identified that the UK Biobank PRS for snoring was reproducible in the Korean cohort and that genetic risk served as an independent risk factor for snoring in the Korean population. These findings may help to develop personalized approaches to reduce snoring in individuals with high genetic risk.

## Introduction

Snoring is a respiratory sound (or noise) that originates during sleep and can be nocturnal or diurnal. Snoring is typically an inspiratory sound, although there may be a small expiratory component, particularly in patients with obstructive sleep apnea, with different spectral features^1^. The prevalence of snoring is often debated, but at least 30% of adults and potentially up to 50% of certain demographic groups snore^2^. A survey of 5713 American residents identified habitual snoring in 24% of men and 13.8% of women. This increased to 60% of men and 40% of women aged 60–65 years, suggesting an age-related increased susceptibility to snoring^3^.

While lifestyle factors are recognized as risk factors for snoring, including obesity, male sex, and aging, the role of genetics in snoring remains unclear^4–8^. Previous studies have explored the potential genetic factors involved in snoring and sleep apnea, including twin and familial studies^4,9–12^. Twin studies have confirmed the genetic predisposition to snoring by demonstrating more consistent snoring-related characteristics in identical twins compared to fraternal twins, with relatively fewer external factors in the correlation between identical twin characteristics^12^. Family studies have shown that people with a family history of snoring are more likely to snore^11^. However, some genetic possibilities can be mediated by other lifestyle factors, such as smoking and alcohol consumption, which can also contribute to snoring^13,14^.

One way to measure the impact of genetic risk is through the use of a polygenic risk score (PRS). PRS has been widely used to estimate genetic risks for various diseases, such as cardiometabolic diseases, by aggregating many single-nucleotide polymorphisms identified through genome-wide association studies (GWAS)^15^. In this study, we aimed to investigate whether genetics plays an independent role in snoring after adjusting for lifestyle factors. Given that the effect of polygenic risks may differ across ethnic groups, we calculated the PRS for snoring from the UK Biobank^1^ and applied it to a Korean cohort^2^. We aim to reproduce the snoring PRS found in the white British or white European portion of the UK Biobank and determine whether genetics serves as an independent risk factor in the Korean population. Additionally, we aimed to investigate the interaction of lifestyle factors and genetic risk on snoring. Our purpose was to investigate whether a PRS for snoring (developed in UK Biobank) is associated with greater odds of snoring in the Korean population, and then to further this idea by including lifestyle-related covariates and investigating the interaction of lifestyle factors and genetic risk on snoring.

## Methods

### Study population

This was a retrospective cross-sectional study. The study population was derived from adults aged > 40 years from the Ansan and Ansung cohort study, a part of the Korean Genome Epidemiology Study (KoGES)^16^. Epidemiological and clinical information was collected through questionnaires and examination after obtaining consent from the participants.

As this study's target phenotype, snoring was assessed as a single question: *"Have you ever heard that you snore?*" This survey question could be answered with “*Yes*”, “*No*”, or “*Prefer not to answer.*” We excluded participants whose answers were “*Prefer not to answer*” (n = 28) from our analyses.

The participants' general information included sex, age, alcohol consumption, smoking behavior, body mass index (BMI), physical activity calculated as metabolic equivalent of task-minute per week (MET) and sleeping time. After determining the MET value for each activity item by referring to previous studies^17,18^, the average MET value for each activity was calculated and then multiplied by the weekly activity hours to calculate MET-hour/week. The survey questions related to smoking and alcohol consumption were as follows: “*Do you normally abstain from drinking alcohol, or have you never consumed alcohol (for religious or other reasons)?*” and “*Do you currently smoke cigarettes?*”. These questions were coded as categorical binary variables.

### Genetic data analysis and quality control

Genotyping of the study data was performed using the KoGES Korean Chip Array. The samples were excluded based on the following criteria: (1) a call rate below 97%, or potential high heterozygosity suggesting sample contamination, (2) over 15 single nucleotide polymorphisms (SNPs) present in only one sample, (3) sex discrepancy, (4) cryptic first-degree relatives, and (5) withdrawals and blind replicates. SNPs were excluded based on the following criteria: exclude all low-quality SNPs in any batch, Hardy–Weinberg equilibrium (pHWE ≥ 5 × 10^−6^), and call rate less than 95%. Imputation for autosomal variants was performed using Eagle v2.3 and IMPUTE4 using a reference panel constructed from 1000 Genomes Project Phase 3, and the Korean reference genome (397 samples) was used as reference panels. Postimputation filtering was used to exclude variants with INFO < 0.8 and MAF < 1%.

### Generation of polygenic risk scores

We calculated PRS based on genome-wide summary statistics for snoring from European population studies [PMID: 32060260]^8^. The PRS (called PRS-Campos) was proposed and validated by Campos et al.^8^, and is based on summary statistics from a large-scale GWAS of snoring.

To compare the performance of PRS-Campos, the PRS of Korean ancestry (PRS-KoGES) was calculated using PRSice and PRS based only on genome-wide significant SNPs from discovery samples (same discovery sample as for PRS-Campos [approximately 408,000 samples] and KoGES samples [approximately 5465 samples]).

PRS-KoGES was calculated, evaluated, and plotted using PRSice software^19^. This software generates a PRS by summing all trait-related alleles in the target sample, weighted by the effect size of each allele in the underlying GWAS^20^. Linkage disequilibrium (LD) aggregation and p value thresholds were used to select the optimal set of trait-related alleles. Imputed base SNPs were filtered with information scores < 0.9 and MAF < 0.01. In addition, the attributed target SNPs were filtered using an information score of < 0.9. SNPs in LD were grouped such that no additional weight was assigned to a single marker. The most representative SNP with the smallest p value was selected within a 250 kb window with r^2^ > 0.1. PRS-KoGES was generated from a gradually increasing p value threshold in the default GWAS. The optimal threshold was selected to account for the largest variation in the target sample.

Previous research indicates that PRSice is well suited to detect the cumulative effect of SNPs in target sample sizes of at least 100 subjects and base sample sizes of at least 50,000 subjects^19^. We calculated the PRS-KoGES in our Korean population cohort (N = 5465) using summary statistics from a large-scale GWAS (N = 408,317).

To calculate PRS in the Korean data, we first examined 42 snoring-associated SNPs that were previously established based on the European study population [PMID: 32060260]^8^. Among the 42 SNPs, 28 were identified in the Korean SNP chip data, and of the 28 SNPs, 20 SNPs showed the same direction of effect between the European and Korean chip data. However, eight SNPs showed the opposite direction of effect between the European and Korean chip data (Supplementary Table 1). The rs592333 SNP on the DLEU7 gene showed a p value of less than 0.05, and the others were not significant in Korean SNP chip data. That is, the 28 SNPs were genome-wide significant in the European chip data; however, these SNPs were not genome-wide significant in the Korean population, and the magnitude of effect for the SNPS that had the same direction as the Europeans was also not similar.

We calculated the variance described by the PRS using the 20 SNPs present in the Korean data. When using the effect size (odds ratio for snoring) of European data, the variance explained (Nagelkerke R2) was 0.5403%. The PRS for snoring was significantly associated with recent snoring for all but one (p ≤ 5e−14) of the p value inclusion thresholds (Supplementary Fig. 1).

### Assessment of lifestyle variables and interactions

In this study, smoking, drinking, physical activity, and late sleep time were selected as variables that reflect an individual's lifestyle. To investigate the relationship between snoring and lifestyle, we only included variables related to modifiable lifestyle habits while excluding uncontrollable variables such as family history to present clear results. In particular, we named those covariates lifestyle factors in this study. We assessed whether these lifestyle factors mitigated the genetic risk of developing snoring. To evaluate this, we focused on the highest (top 20%) vs. lowest genetic risk quintiles (bottom 20%), as the greatest genetic risk/protection is at the extremes of risk^21^. This analysis investigated the relationship between lifestyle and polygenic risks and their tendency to snore. The odds ratio of high genetic risk to low genetic risk in lifestyle was calculated in the high- and low-lifestyle-risk groups.

### Statistical analysis

Phenotype-derived estimates, such as prevalence and associations between variables and stratified plots of snoring prevalence, were performed using R. In the study population, the differences in the statistics of covariates according to snoring were tested. In this case, if the data type of the covariate was categorical, the prevalence and proportion were used as statistics, and the difference according to phenotype expression was tested using the chi-squared test. In contrast, if the data type was continuous, the mean and standard deviation were used as statistics, and differences according to phenotype expression were tested using the t test. Additionally, the demographic characteristics of the KoGES population and those of the UK Biobank were compared and tested for differences.

Using lifestyle factors, the risk ratio for snoring was expressed as an odds ratio (OR). Crude OR, which treated each covariate as a univariate risk factor, and adjusted OR, which treated each covariate as a multivariate risk factor, were both calculated. Both ORs are shown through a forest plot. The explanatory power of the multivariate logistic regression model was estimated using the R-squared value. Based on the estimated coefficient, we presented ORs in this study.

### Ethical approval and informed consent

This study was performed in accordance with the relevant guidelines and regulations of the Seoul National University Bundang Hospital Institutional Review Board (SNUBH IRB). Exemption from deliberation is reasonable in accordance with Articles 13 and 33 of the Enforcement Regulations of the Bioethics and Safety Act and HRPP SOP II.A.3. According to the research plan, it is judged to be a study that collects and uses genetic information data and survey data from Anseong/Ansan cohort, and it is not judged that the research is provided with human materials. Since the data in this study are non-identification data that do not contain personal identification information, this study was approved based on waivers of informed consent or exemptions by the SNUBH IRB (SNUBH IRB No: B-1805-471-301, X-2112-727-901).

## Results

### General characteristics of the study population

The total number of participants in the KoGES cohort was 5465 (male:female = 2604:2861). The mean age of the participants was 51.7 years (SD 8.3). Snoring was reported in 3526 (64.5%) of 5465 participants.

Table 1 shows the general characteristics of the snorers and nonsnorers. In total, 52% of snorers were identified as male, and 60% of nonsnorers were female. There was a significant difference in the mean age between the snoring and nonsnoring groups. There were also significant differences (p < 0.001) in drinking and smoking habits between the two groups. In total, 57% of snores had *a history of alcohol consumption*, while 48% of nonsnorers reported drinking behavior. Regarding smoking behavior, approximately 44% of snorers and 33% of nonsnorers were smokers, showing significant differences (p < 0.001). There was also a significant difference (p < 0.001) in BMI between these groups, and the BMI of the snorers was 25.1 (kg/m^2^), and that of the nonsnorers was 23.9 (kg/m^2^). There was no significant difference in the MET (p = 0.313) and mid-sleep time (p = 0.06) between the snoring and nonsnoring groups.

**Table 1:** General characteristics of KoGES cohort according to snoring.

Characteristics	Snorers (N = 3526)	Non-snorers (N = 1939)	p value
Sex (male), N (%)	1828 (52%)	776 (40%)	< 0.001^a)^
Age (years), mean (SD)	51.7 (8.32)	51.28 (8.82)	< 0.001^b)^
Drinking behavior, N (%)	2020 (57%)	936 (48%)	< 0.001^a)^
Smoking behavior, N (%)	1517 (44%)	656 (33%)	< 0.001^a)^
Body Mass Index (kg/m^2^), mean (SD)	25.06 (3.05)	23.85 (2.88)	< 0.001^b)^
MET^†^ (min/week), mean (SD)	1410 (1036.61)	1440 (1058.05)	0.313^b)^
Sleep mid-time after 2 AM, N (%)	2511 (71%)	1331 (69%)	0.06^a)^

^a)^Chi-squared test used, ^b)^ t-test used, ^†^metabolic equivalent of task.

### Differences in the distribution of population groups according to Korean and European races

Table 2 shows the differences in sex, age, and BMI distribution between the UK and Korean populations. The sex distribution was significantly different, with more females in the Korean snoring group (p = 0.045). The average age of Korean snorers was 51.7 years and that of snoring in the UK Biobank was 57.01 years, indicating a significant difference (p < 0.001). BMI was 25.06 (kg/m^2^) for Koreans and 28.67 (kg/m^2^) for Europeans, showing a significant difference between the two groups (p < 0.001).

**Table 2:** Sex, age, and BMI distribution differences between the UK and Korea.

	Cases (snorers)	Controls	Total	p value
Female, N (%)				
KoGES	1698 (48%)	1163 (60%)	2861 (52%)	0.045^a)^
UK Biobank	63,833 (40.74%)	161,775 (61.44%)	225,608 (53.72%)	
Age, mean (SD)				
KoGES	51.7 (8.32)	51.28 (8.82)	51.55 (8.5)	< 0.001
UK Biobank	57.01 (7.70)	56.60 (8.21)	56.75 (8.03)	
BMI (kg/m^2^), mean (SD)				
KoGES	25.06 (3.05)	23.85 (2.88)	24.63 (3.04)	< 0.001
UK Biobank	28.67 (4.85)	26.64 (4.52)	27.39 (4.75)	

^a)^Chi-squared test used.

### Risk factors associated with snoring

Table 3 summarizes the relationship between snoring and PSG parameters. The adjusted odds ratio accounts for age and sex when evaluating the association between PRS and snoring. To understand the risk factors for snoring and evaluate the independent association between snoring and each risk factor, we estimated the overall explanatory power of the risk factor as an R-squared value by using both genomic and life factors as covariates (Supplementary Table 2).

**Table 3:** Logistic regression results of polygenic risk factors for snoring.

Covariates	Crude OR		Adjusted OR	
	OR	p value	OR	p value
PRS	1.19 (1.09–1.3)	< 0.001***	1.18 (1.08–1.29)	< 0.001***

The values are presented for ‘non-snorers’ versus ‘snorers’ groups.

Significance codes: 0 < *** < 0.001 < ** < 0.01 < * < 0.05.

The highest crude odds ratio for snoring was higher BMI (OR 1.96, 95% CI 1.74–2.19), followed by male sex (OR = 1.61, 95% CI 1.09–1.3), smoking behavior (OR 1.48, 95% CI 1.32–1.66), drinking behavior (OR 1.44, 95% CI 1.29–1.61]), polygenic risks such as higher PRS (OR 1.19, 95% CI 1.09–1.3), late sleep mid-time (OR 1.12, 95% CI 0.99–1.27), older age (OR 1.11, 95% CI 0.99–1.24), and lower physical activity (OR 0.91, 95% CI 0.99–1.27). In addition, the highest adjusted odds ratio for snoring was higher BMI (OR 1.98, 95% CI 1.76–2.23), followed by male sex (OR 1.54, 95% CI 1.28–1.86), older age (OR 1.23, 95% CI 1.03–1.35), higher PRS (OR 1.18, 95% CI 1.08–1.29), drinking behavior (OR 1.18, 95% CI 1.03–1.35), late sleep mid-time (OR 1.17, 95% CI 1.02–1.33), smoking behavior (OR 0.99, 95% CI 0.82–1.19), and lower physical activity (OR 0.92, 95% CI 0.85–1.00).

**Figure 1:**
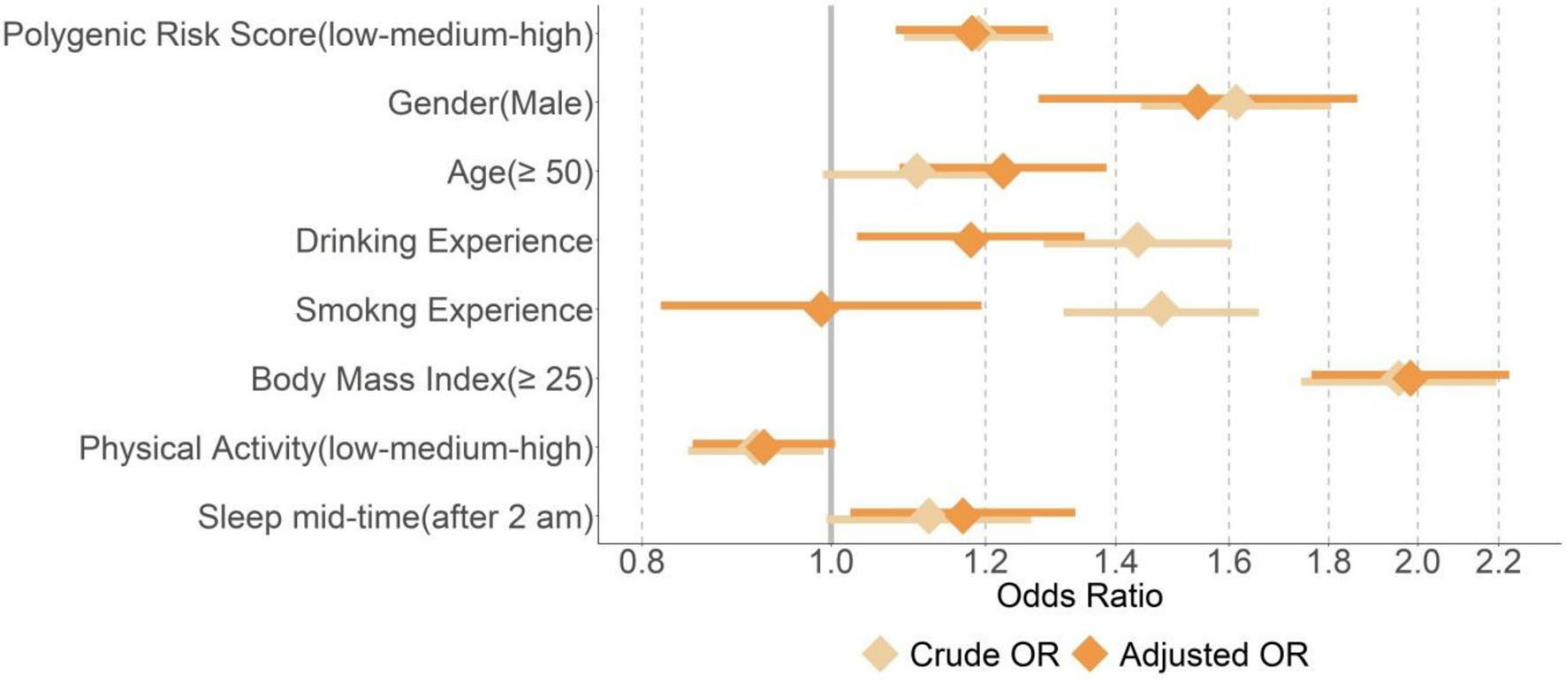
Forest plot depicting the odds ratios of studied variables on snoring.

Figure 1 shows a forest plot depicting the odds ratios of each variable for snoring. Crude OR, which treated each covariate as a univariate risk factor, and adjusted OR, which treated each covariate as a multivariate risk factor, are shown. The smoking variable showed the largest difference between the univariate analysis (OR 1.48, 95% CI 1.32–1.66) and the multivariate analysis (OR 0.99, 95% CI 0.82–1.19). In contrast, PRS and physical activity showed little difference between univariate analysis (OR 0.91, 95% CI 0.84–0.99) and multivariate analysis (OR 0.92, 95% CI 0.85–1.00).

### Interaction between PRS and lifestyle factors

**Figure 2:**
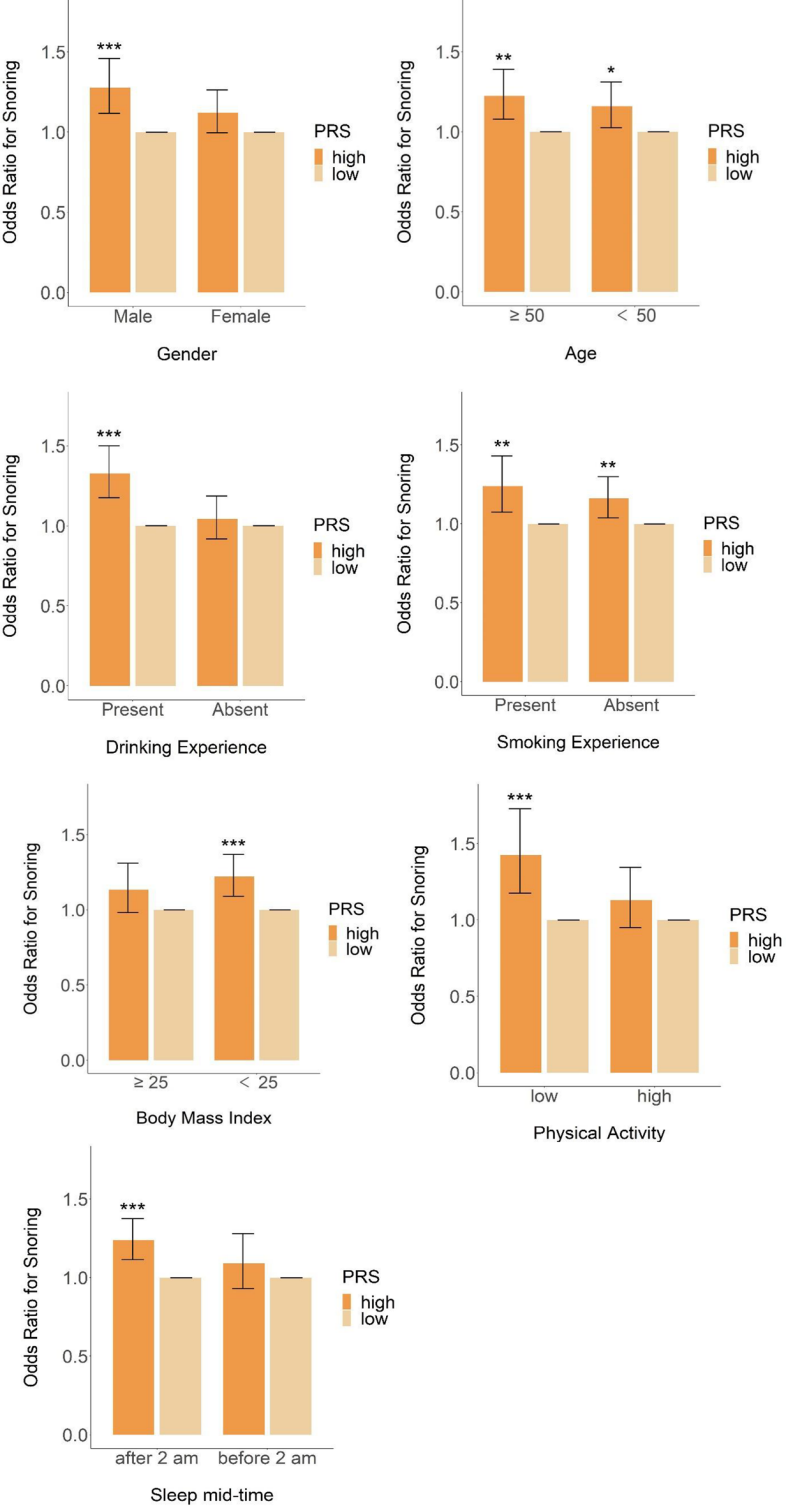
Interaction plot.

The results of the interaction analysis between the PRS and lifestyle variables are shown in Table 4 and Fig. 2. The odds ratio of the PRS-low group was set to 1 for comparison. In the high lifestyle risk group, the variable with the highest odds ratio (OR 1.43, 95% CI 1.18–1.73) was physical activity (p < 0.001), followed by drinking behavior (OR 1.33, 95% CI 1.17–1.50, p < 0.001) and sex (OR 1.28, 95% CI 1.12–1.46, p < 0.001), and there was a significant difference in level. BMI showed no significant difference in odds (OR 1.14, 95% CI 0.98–1.31, p = 0.086). In contrast, in the low lifestyle risk group, the odds ratios of BMI (OR 1.222, CI = 1.09–1.37, p < 0.001), smoking behavior (OR 1.16, CI 1.04–1.30, p = 0.009), and age (OR 1.12, CI 1.00–1.26, p = 0.018) showed significantly different results.

**Table 4:** Interaction analysis for snoring between PRS and lifestyle variables.

Covariates	High lifestyle risk group		Low lifestyle risk group	
	OR for PRS high to low	p value	OR for PRS high to low	p value
Sex	1.28 (1.12–1.46)	< 0.001***	1.12 (1.00–1.26)	0.057
Age	1.22 (1.08–1.39)	0.002**	1.16 (1.03–1.31)	0.018*
Drinking behavior	1.33 (1.17–1.50)	< 0.001***	1.04 (0.92–1.19)	0.522
Smoking behavior	1.24 (1.07–1.43)	0.003**	1.16 (1.04–1.30)	0.009**
Body Mass Index	1.13 (0.98–1.31)	0.086	1.22 (1.09–1.37)	< 0.001***
Physical activity	1.43 (1.18–1.73)	< 0.001***	1.13 (0.95–1.35)	0.166
Sleep mid-time	1.24 (1.11–1.38)	< 0.001***	1.09 (0.93–1.28)	0.286

Significance codes: 0 < *** < 0.001 < ** < 0.01 < * < 0.05.

## Discussion

Although snoring is common in the general population, it has been largely understudied from an individual genetic and lifestyle perspective. In particular, the clinical relevance of the PRS for snoring has not been fully elucidated in Asian populations. In this study, we calculated the most recent PRS for snoring and (1) showed the difference explained by the PRS between UK Biobank participants of European ancestry and Korean participants of Asian ancestry and (2) analyzed its relationship with lifestyle factors such as smoking and alcohol use, physical activity, and sleeping time features to investigate the degree to which these individual factors modify the inherited susceptibility to snoring. To investigate the relationship between snoring and lifestyle, we only included variables related to modifiable lifestyle habits while excluding uncontrollable variables such as family history to present clear results.

An early cohort study on the genetic characteristics of snoring was conducted in a cardiovascular disease study cohort consisting of 3387 men aged 54–74^9^ years. A total of 3308 participants answered the survey, and habitual snoring strongly correlated with the family history of grandparents, parents, brothers, and children. The largest difference between the group that complained of habitual snoring and the control group was the family history of self-reported snoring. Another previous study reported genetic results on snoring (n = 408,000; snorers = 152,000) using data from the UK Biobank^8^. In total, 37% of all study subjects had snoring, and snorers had a higher rate of diagnosed sleep apnea than subjects without snoring (2.88% of snoring patients vs. 0.63% of controls). Snoring was correlated with age and sex and showed a positive correlation with BMI, smoking, and alcohol intake frequency and a negative correlation with socioeconomic status. They identified 42 significant genome-wide loci with an SNP-based heritability estimate of approximately 10% on the liability scale. Genetic correlations with body mass index, alcohol intake, smoking, schizophrenia, anorexia nervosa, and neuroticism were observed in the European population. Polygenic scores predicted recent snoring and probable obstructive sleep apnea in an independent Australian sample (n = 8000)^22^. A potential causal relationship between high BMI and snoring was suggested based on Mendelian randomization analysis results.

Several studies have presented comparisons of PRSs by applying genetic analysis of specific phenotypes to other independent group data^20,23–27^. According to a previous study that assessed PRSs for coronary artery disease and type 2 diabetes as predictive factors for cardiovascular (CV) mortality^24^, both CAD PRSs were significantly correlated with CV mortality risk. These associations remained significant even after adjusting for a wide range of demographic and clinical characteristics. Adherence to an unhealthy lifestyle was also significantly linked to an elevated risk of CV mortality in the very high genetic risk group (favorable vs. unhealthy lifestyle with very high genetic risk for CAD PRS. In all genetic risk categories, the population attributable fraction (PAF) for CV mortality was 32.1%, while the PAF for smoking was 14.1%. Age, sex, and lifestyle factors did not significantly interact with PRSs predicting the risk of CV mortality.

Another study included a comprehensive health checkup database from the Korean population in conjunction with genotyping to generate PRS for BMI^27^. This study included a phenome-wide association (PheWAS) analysis, and a longitudinal association between BMI and PRS-BMI was observed. A model that predicted 10-year BMI based on age, sex, and baseline BMI was more accurate after including PRS-BMI. Higher deciles of PRS were directly correlated with changes in BMI in a linear mixed model evaluating longitudinal changes in BMI with age. Significant correlations were found between PheWAS and metabolic syndrome, bone density, and fatty liver disease. A study identified several genetic loci associated with sleep apnea traits, such as the apnea–hypopnea index and nocturnal hypoxemia, among Hispanics and Latino Americans^28^. Although not specifically focused on genomic factors, this review highlights the long-term health consequences of untreated obstructive sleep apnea, emphasizing the importance of understanding the underlying genetic factors^29^.

In our study, the genetic snoring risk score was calculated for 3526 snorers and 1939 nonsnorers by applying the PRS based on the snoring GWAS results of a European study. Because of the analysis of the various stages of significance, the GWAS p value cutoff showed the highest R2 (0.5403%). Nevertheless, it did not reach the explanatory power of the previous study. This means that the genetic explanatory power of the snoring GWAS did not reach that of the snoring PRS calculated in the evaluation group of the same ethnic group.

Overall, the results of our study suggest that the odds ratio for snoring in the PRS high group was high. Thus, the effect of PRS can be interpreted as a genetic risk factor. Lifestyle variables interpreted as having genetic risk factors were alcohol consumption, sleeping late (derived by sleeping mid-time), and smoking. In the case of BMI, individuals with a low BMI are less likely to snore which seems to be due to genetic influences. Our results showed that the odds of snoring were high when exposed to risk factors such as PRS, sex, age, drinking behavior, BMI, and sleep duration. The PRS appeared to interact with other factors associated with snoring, such as male sex, older age, alcohol consumption, smoking, lower BMI, low physical activity, and late sleep mid-time. Our research results are presented based on the odds ratio concerning lifestyle habits and snoring. SNPs with genetic significance in previous papers are presented as a supplementary table. However, these SNPs were not significant in the Korean population.

While the underlying biological mechanism linking low BMI to an increased odd of snoring is not yet fully understood, it is thought to be related to upper airway obstruction resulting from craniofacial abnormalities, which may be more commonly associated with polygenic risks. Our study has shown that individuals with a low BMI may have a higher PRS for snoring. In addition, BMI showed high odds when comparing the adjusted results in multivariate analysis. This indicates that there was no significant change in the odds ratio even with the same genetic influence, which may also indicate that lifestyle habits are important factors for snoring.

To the best of our knowledge, this is the first study to investigate the associations between lifestyle habits and the genetic risk of snoring in the Korean population derived from European PRSs. To date, most large-scale genetic studies have been conducted in European populations. However, in the case of snoring, the effect size (odds ratio for snoring) was very small, with an odds ratio of 0.99–1.01, as seen in the European GWAS results. Hence, a large-scale cohort study is required to develop other ethnicity, including the Korean PRS model. The aim of this study was to apply the snoring PRS from European cohorts to a Korean cohort and to examine whether the results are reproducible.

The limitations of this study are as follows. First, there were no Korean snoring GWAS data that could be applied to the PRS model in this study. Therefore, it was impossible to conduct a preliminary analysis to verify the difference in the basic genetic structure of the European group used as a reference and the Korean group. Second, the explanatory power may have decreased because of the small sample size. Third, since this study was conducted using the KoGES^16^ cohort data in Korea, the snoring phenotype was determined based on the self-reported snoring question, which is not a standardized question. Snoring was assessed as a single item (Field-ID: 1210) in a UK Biobank survey question: “*Does your partner or a close relative or friend complain about your snoring?*” Meanwhile, snoring was assessed in KoGES cohort data by the question: "*Have you ever heard that you snore?*" Both of these questions were answered with “*Yes*”, “*No*”, “*Don’t know*”, or “*Prefer not to answer*”. Although the snoring question of the KoGES data has been used in various sleep association studies, including carotid atherosclerosis^30^, renal function^31^ and hypertension^32^, this can be one of our limitations.

## Conclusion

Here, we used data from a Korean adult sample to assess the prevalence of snoring in the community and track its associations with lifestyle-related factors. To determine the extent to which these sociodemographic and lifestyle factors affect the inherited propensity to snore, we calculated the most recent PRS for snoring and (1) displayed the difference explained by the PRS between European and Korean adults and (2) analyzed its relationship with lifestyle factors such as smoking and alcohol consumption, physical activity, and sleeping features. Given the small effect size of the SNP-based association, PRS serves as an excellent diagnostic and therapeutic marker in clinical settings. Therefore, it is vital to verify the causal relationship or genetic association between characteristics by utilizing an analytical approach such as GWAS for various components known as risk factors for snoring as a method for analyzing polygenic risks.

### Supplementary Information

Supplementary Information.

## Data Availability

The KoGES (Korean Genome and Epidemiology Study) genotype data that support the findings of discovery stage are available upon request under data sharing policy of National Research Institute of Health, Korea (http://www.nih.go.kr/). Other data that support our findings are available from the corresponding author upon reasonable request.
