# Supplementary material for: Snoring-related polygenic risk and its relationship with lifestyle factors in a Korean population: KoGES study
Source: Sci Rep. 2023 Aug 30;13:14212. doi: 10.1038/s41598-023-41369-x (PMC10469207; doi:10.1038/s41598-023-41369-x)

### Supplementary materials

Supplementary Table 1. Comparison of PRS results between UK Biobank and Korean study.

| Direction of allelic effect compared to UK biobank | Top prioritized  mapped gene | rsID | effect  allele | UK  MAF | KoGES  MAF | UK  OR | KoGES  OR | UK  p-value | KoGES  p-value |
| --- | --- | --- | --- | --- | --- | --- | --- | --- | --- |
| the same | DLEU7 | rs592333 | A | 0.442 | 0.215 | 0.991 | 0.895 | 1E-17 | 2E-02 |
|  | BCL11B | rs2664299 | T | 0.415 | 0.949 | 1.008 | 1.067 | 1E-12 | 5E-01 |
|  | TRAM1 | rs13251292 | A | 0.415 | 0.889 | 0.993 | 0.923 | 4E-12 | 2E-01 |
|  | RP11-42L9.2 | rs725861 | A | 0.192 | 0.762 | 0.991 | 0.972 | 1E-11 | 5E-01 |
|  | FTO | rs796856741 | G | 0.443 | 0.842 | 0.993 | 0.978 | 5E-11 | 7E-01 |
|  | LINC00332 (lincRNA) | rs12429765 | A | 0.493 | 0.555 | 1.007 | 1.042 | 6E-11 | 3E-01 |
|  | AC105242.1 (miRNA) | rs7829639 | A | 0.297 | 0.21 | 0.993 | 0.967 | 1E-10 | 5E-01 |
|  | SKAP1:RP11-456D7.1 | rs11409890 | T | 0.482 | 0.704 | 1.007 | 1.047 | 2E-10 | 3E-01 |
|  | SMG6 | rs8069947 | C | 0.488 | 0.693 | 1.007 | 1.09 | 3E-10 | 4E-02 |
|  | ROBO2 | rs74936745 | AC | 0.444 | 0.577 | 1.007 | 1.12 | 4E-10 | 6E-03 |
|  | RNA5SP471 | rs8108822 | C | 0.102 | 0.845 | 1.011 | 1.018 | 6E-10 | 8E-01 |
|  | VKORC1 | rs59502288 | G | 0.365 | 0.927 | 0.993 | 0.907 | 9E-10 | 2E-01 |
|  | Y_RNA (misc_RNA) | rs9900496 | T | 0.317 | 0.601 | 0.993 | 0.978 | 2E-09 | 6E-01 |
|  | PTGES3 | 12:57088077_CA_C | CA | 0.305 | 0.267 | 1.007 | 1.071 | 3E-09 | 1E-01 |
|  | RP11-183G22.1 (pseudogene) | 1:50819256_CA_C | CA | 0.492 | 0.12 | 1.006 | 1.116 | 4E-09 | 8E-02 |
|  | SIM1 | rs17060460 | A | 0.235 | 0.329 | 0.993 | 0.962 | 1E-08 | 4E-01 |
|  | CEP120 | rs34732995 | C | 0.476 | 0.394 | 1.006 | 1.02 | 2E-08 | 6E-01 |
|  | MACF1 | rs80093081 | T | 0.292 | 0.309 | 1.006 | 1.058 | 2E-08 | 2E-01 |
|  | SNAP91 | rs2207944 | T | 0.457 | 0.397 | 0.994 | 0.982 | 2E-08 | 7E-01 |
|  | LMO4 | 1:87773720_CT_C | CT | 0.338 | 0.757 | 1.006 | 1.02 | 3E-08 | 7E-01 |
| the opposite | MSRB3 | rs10878269 | C | 0.35 | 0.575 | 0.991 | 1.028 | 2E-16 | 5E-01 |
|  | POC5 | rs2307111 | T | 0.396 | 0.431 | 1.008 | 0.981 | 5E-13 | 6E-01 |
|  | C5orf66 | rs4976269 | G | 0.341 | 0.818 | 1.007 | 0.955 | 9E-10 | 4E-01 |
|  | RP5-859D4.3 | rs6054427 | G | 0.378 | 0.124 | 0.994 | 1.005 | 4E-09 | 9E-01 |
|  | NSUN3 | rs202110996 | A | 0.394 | 0.607 | 1.006 | 0.963 | 5E-09 | 4E-01 |
|  | U3 (snoRNA) | rs145367119 | C | 0.431 | 0.495 | 1.006 | 0.956 | 1E-08 | 3E-01 |
|  | KCNQ5 | rs947612 | G | 0.229 | 0.757 | 1.007 | 0.993 | 2E-08 | 9E-01 |
|  | RNU6-929P | rs6099273 | C | 0.235 | 0.806 | 0.993 | 1.005 | 3E-08 | 9E-01 |

Supplementary Table 2. Logistic regression results of potential risk factors for snoring.

The values are presented for ‘non-snorers’ versus ‘snorers’ groups.

| Covariates | Crude OR | | Adjusted OR | |
| --- | --- | --- | --- | --- |
|  | OR | P-value | OR | P-value |
| PRS | 1.19 (1.09-1.3) | <0.001 *** | 1.18 (1.08-1.29) | <0.001 *** |
| Gender | 1.61 (1.44-1.81) | <0.001 *** | 1.54 (1.28-1.86) | <0.001 *** |
| Age | 1.11 (0.99-1.24) | 0.073 | 1.23 (1.08-1.38) | 0.001 ** |
| Drinking Experience | 1.44 (1.29-1.61) | <0.001 *** | 1.18 (1.03-1.35) | 0.016 * |
| Smoking Experience | 1.48 (1.32-1.66) | <0.001 *** | 0.99 (0.82-1.19) | 0.905 |
| Body Mass Index | 1.96 (1.74-2.19) | <0.001 *** | 1.98 (1.76-2.23) | <0.001 *** |
| Physical Activity | 0.91 (0.84-0.99) | 0.029 * | 0.92 (0.85-1.00) | 0.064 |
| Sleep mid-time | 1.12 (0.99-1.27) | 0.061 | 1.17 (1.02-1.33) | 0.022 * |

Significance codes: 0<***<0.001<**<0.01<*<0.05

Explanatory Power: Nagelkerke pseudo R2 value is 5.98%

Goodness of fit of the model: The likelihood-ratio test's p-value is<0.001, so this model has statistical significance.

Autocorrelation check: The Durbin-Watson test's p-value is 0.284, which is larger than 0.05; therefore, there is no autocorrelation multicollinearity check: the VIF values of each covariate are less than 3, and there is no multicollinearity.

Supplementary Figure 1. PRS bar plot.


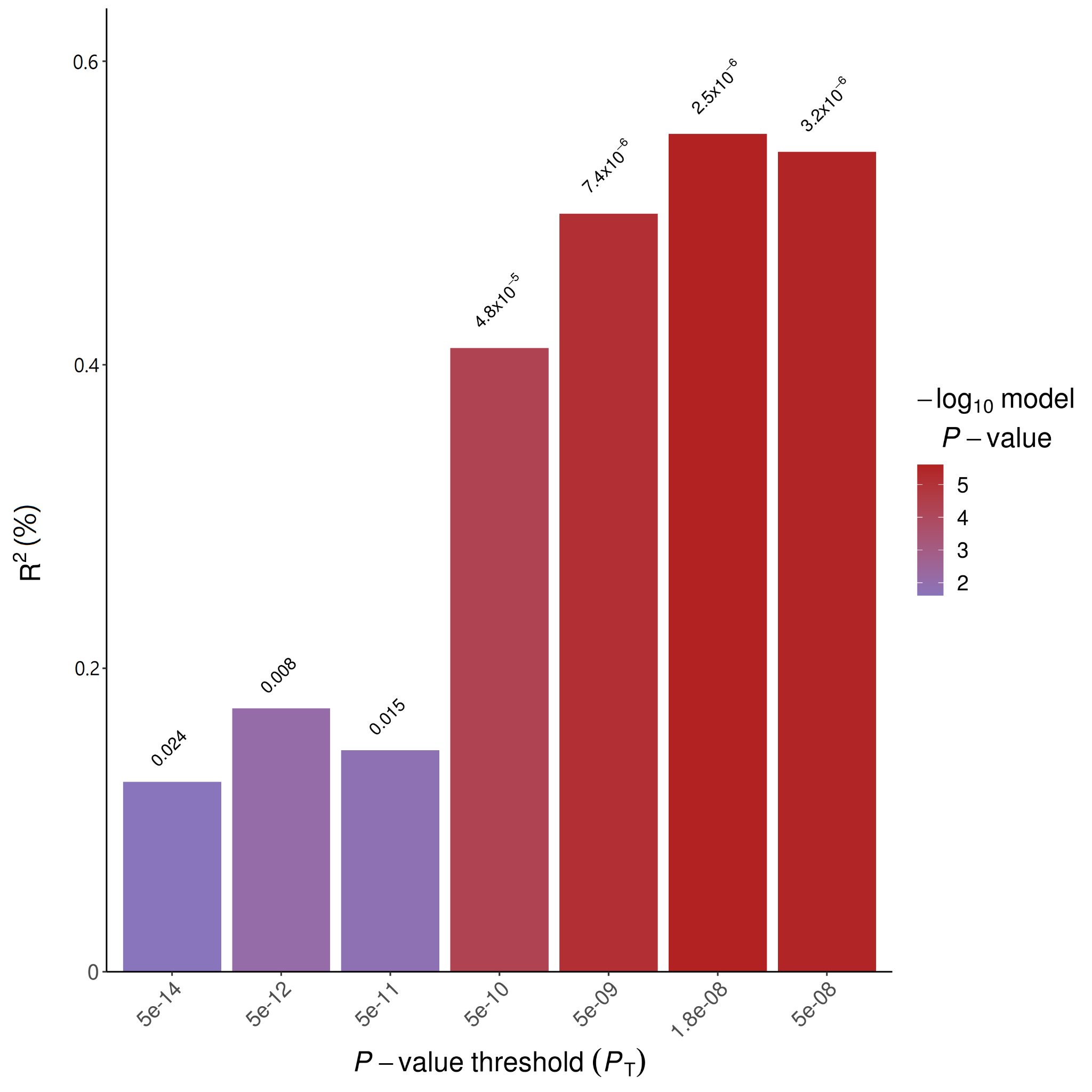

Supplement: Supplementary file 1 — Supplementary Information. [file 41598_2023_41369_MOESM1_ESM.docx]
